# Endovascular treatment of portal hypertension and recurrent digestive hemorrhage secondary to arterioportal fistula syndrome: late complication of penetrating abdominal trauma

**DOI:** 10.1590/1677-5449.190136

**Published:** 2020-04-03

**Authors:** Matheus Bertanha, Regina Moura, Rodrigo Gibin Jaldin, Marcone Lima Sobreira, Arthur Curtarelli, Felipe Damacena Rosa, Marcelo Sembenelli, Winston Bonetti Yoshida

**Affiliations:** 1 Universidade Estadual Paulista “Júlio de Mesquita Filho” – UNESP, Cirurgia e Ortopedia, Botucatu, SP, Brasil.; 2 Universidade Positivo, Faculdade de Medicina, Curitiba, PR, Brasil.

**Keywords:** vascular fistula, arteriovenous fistula, portal hypertension, penetrating wounds, endovascular procedures

## Abstract

The arterioportal fistula (APF) syndrome is a rare and reversible cause of pre-sinusoidal portal hypertension, caused by communication between a visceral artery and the portal venous system. Most patients are asymptomatic, but when they do develop symptoms, these are mainly related to gastrointestinal bleeding, ascites, congestive heart failure, and diarrhea. This therapeutic challenge presents a case of APF caused by a 20-year-old stabbing injury with unfavorable late clinical evolution, including significant malnutrition and severe digestive hemorrhages. The patient was treated using an endovascular procedure to occlude of the fistula.

## INTRODUCTION

The arterioportal fistula syndrome (AFS) is a rare condition that is often linked with abdominal traumas and consists of abnormal communication between a visceral artery and the portal venous system, causing presinusoidal portal hypertension (PH).^1^ The arteries most often involved are the hepatic artery (65%) and the splenic artery (11%).^2^^-^4

According to Guzman et al.,^1^ AFSs can be classified into three types, depending on etiology, anatomy, and topography:

Type 1: these are small peripheral intrahepatic fistulae with minimal physiological consequences that are generally asymptomatic and progress to spontaneous occlusion within 1 month. They often occur after percutaneous liver biopsies. These fistulae can be followed clinically with Duplex ultrasound (DUS) for 1 month and if they do not occlude within this period and become symptomatic, they should be embolized;
Type 2: these fistulae are larger and more central and can be intrahepatic or extrahepatic. They often occur after penetrating abdominal trauma or due to erosion of an aneurysm of the splenic artery with fistulization into the portal system. They can cause PH, hepatoportal hypertension and hepatic fibrosis. These fistulae should be treated with embolization or conventional surgery (in cases of unsuccessful endovascular treatment or when endovascular techniques are not viable), in order to prevent irreversible complications of PH;
Type 3: congenital fistulae, which are rare, generally intrahepatic and diffuse, and cause severe PH in childhood. Referral to a specialist pediatric center is recommended and the following treatment options are possible: ligature of the hepatic artery, endovascular embolization, hepatectomy, or liver transplantation, depending on the degree of physiological compromise.

The symptomology of APFs is varied and depends on several factors: diameter, topography and, consequently, blood flow to the fistula.^2^^,^^3^ The majority of patients with AFS are asymptomatic, but, when symptomatic, patients can present with upper gastrointestinal bleeding (33%), ascites (26%), congestive heart failure (4.5%), and diarrhea (4.5%).^2^ Diagnosis of APFs can be challenging and arteriography is the examination of choice for confirmation.^1^ Doppler US can be used as a screening examination and is good for follow-up.^1^^,^^5^ Computed tomography angiography (angio-CT) may show premature filling of veins during the arterial phase of the examination and highlight the hepatic arterial phase.^4^^,^^6^ Magnetic resonance angiography can be used in cases in which arteriography is contraindicated.^4^ The ideal treatment for AFS is still controversial. The specific treatment will depend on the size, site, and number of APFs, in addition to the patient’s clinical conditions and the classification of the disease.^7^

## PART I: CLINICAL SITUATION

A 40-year-old male patient was admitted via the emergency room by the clinical gastroenterology team at our institution because of recurrent upper digestive hemorrhage (UDH) and vomiting with a moderate quantity of blood. His clinical history included frequent episodes of melena and anorexia and he also reported progressive weight loss. He was in a poor general state, was pale, and had tachycardia and normal blood pressure. He denied alcoholism and hepatitis (the absence of which was later confirmed by negative serology) or any other hepatic or splenic comorbidities. On physical examination, he was underweight, with a normal abdomen and palpable hepatosplenomegaly. He had a history of trauma, by stabbing, in the left flank 20 years previously. On that occasion he had undergone an exploratory laparotomy, during which only an emergency left nephrectomy had been performed. However, he had developed PH and was later treated with endoscopic ligature of esophageal varices, to control UDH. Approximately 1 year previously, a transjugular intrahepatic portosystemic shunt (TIPS) had been performed at a different institution, but his condition had not improved.

**Figure 1:**
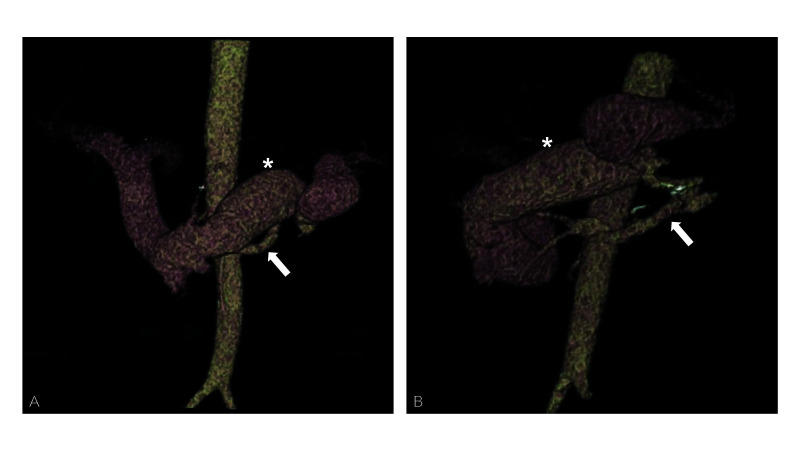
Preoperative angiotomography, venous phase. (A) posteroanterior volumetric rendering, showing the renal artery (arrow) and premature perfusion of the splenic (asterisk), superior mesenteric, and portal veins; (B) the same image with left anterior oblique inclination.

When admitted to our institution, work-up investigation with DUS had detected discrete cirrhosis, a small quantity of free intracavitary liquid, and an enlarged diameter portal vein with elevated splenic flow, suggestive of AFS (probably communication between the remnant left renal artery and the splenic vein). This result was confirmed with angio-CT (Figure 1).

**Figure 2:**
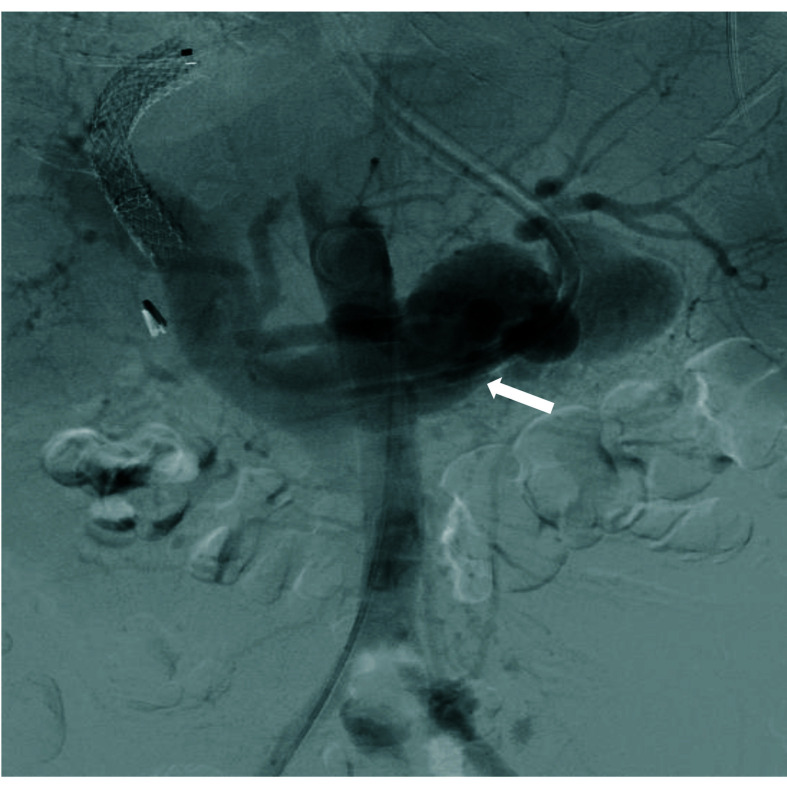
Preoperative aortography showing premature filling of the splenic vein (arrow).

Diagnostic aortography confirmed the AFS between the left renal artery and the splenic vein, with high blood flow and large splenic vein caliber (Figure 2). The AFS was therefore defined as a late type 2, secondary to abdominal trauma. Upper digestive endoscopy identified varicose veins of the esophagus and gastric fundus, with signs of recent bleeding, and ruled out an active peptic ulcer.

In view of this presentation, the following treatment options were considered: conservative clinical treatment; conventional surgical treatment with ligature of the renal artery and splenectomy; endovascular treatment with embolization of the renal artery with detachable coils; endovascular treatment with embolization by deployment of a detachable vascular plug.

## PART II: WHAT WAS DONE

**Figure 3:**
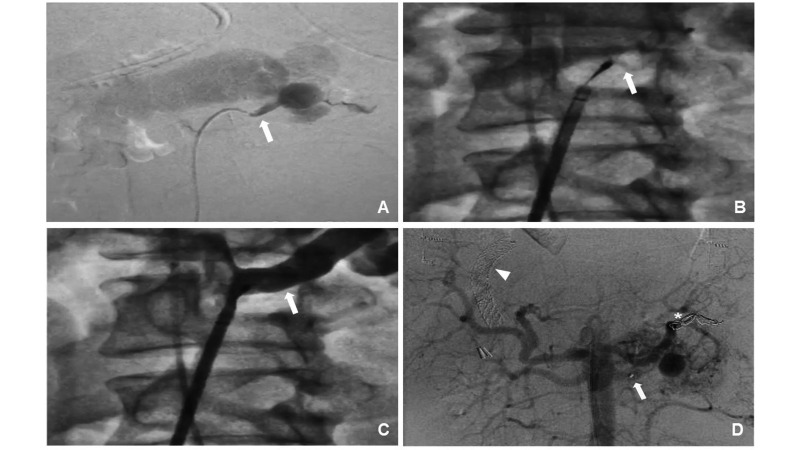
Intraoperative fluoroscopic images. (A) selective arteriography of the left renal artery (arrow) and the fistula with the splenic vein; (B) positioning the vascular plug in the proximal renal artery (arrow); (C) selective arteriography of the renal artery with the vascular plug in place to check its position (arrow); (D) immediate postoperative aortography showing the treatment result, where it is possible to see the plug marked where it has been placed in the renal artery (arrow), free release coils in the splenic artery (asterisk) and the stents placed in a previous intervention for transjugular intrahepatic portosystemic shunt (arrowhead).

The patient underwent endovascular treatment, in which it was decided to occlude the fistula with deployment of a Cera™ vascular plug measuring 14 mm × 10 mm (Lifetech Scientific) in the stump of the left renal artery. Figure 3 shows further details of the treatment, particularly selective catheterization of the renal artery and placement of a long introducer (Destination® 8F 65 cm, Terumo Medical) (Figure 3A), positioning of the vascular plug system (Figure 33C), release, after confirmation of its position, and an image showing the stents used for TIPS, with almost no contrast (Figure 3D).

**Figure 4:**
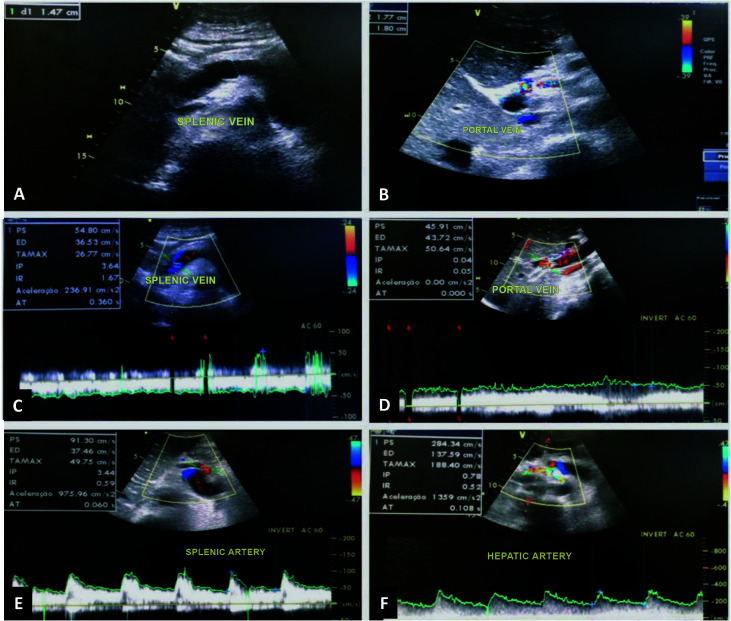
Postoperative follow-up duplex ultrasound (6 months). (A) diameter of the splenic vein is 1.47 cm; (B) diameter of the portal vein is 1.77 cm × 1.80 cm; (C) monophasic venous flow with no signs of fistulization in the splenic vein; (D) monophasic venous flow in the portal vein; (E) normal arterial flow in the splenic artery; (F) normal arterial flow in the hepatic artery.

Since the patient was in the process of digestive hemorrhage because of hypertensive gastropathy, it was difficult to stabilize his hemometric levels even with multiple transfusions and also because he had thrombocytopenia because of hypersplenism, in addition to the principal procedure, the decision was taken to embolize the splenic artery with two 14 cm × 10 mm Nester® free release coils (Cook Medical®), to guarantee that splenic vein output would be reduced, taking into consideration the low rate of complications related to this procedure (Figure 3D). At the end of the procedure, a control angiography showed slow filling of the splenic hilum and total occlusion of the left renal artery, with a significant reduction in the flow of contrast into the portal system (Figure 3D). The patient was kept in hospital for another 5 days to treat anemia and malnutrition. His clinical status improved significantly, he gained weight and was able to resume social and work activities, and is in follow-up with DUS (6 months) with no further episodes of UDH (Figure 4).

## DISCUSSION

A minority of patients with AFS manifest symptoms and those that do present are secondary to hemodynamic and anatomic abnormalities caused by the high vascular flow and increased portal pressure.^1^ Nevertheless, occurrence of PH without evidence of cirrhosis can suggest APFs.^1^ Diagnosis can be made using DUS and later confirmed with angiotomography or selective angiography, which is the standard study for diagnostic confirmation and treatment planning.^8^

The great majority of APFs do not require emergency intervention; there is time to analyze the fistula and estimate its physiological consequences; smaller fistulae may undergo spontaneous thrombosis and, because of this, small asymptomatic peripheral fistulae (classified as type 1) can be followed with serial DUS.^9^ Surgical treatment should be indicated if there is evidence of PH, if the fistula is large, or if it does not disappear within a short period of time.^4^^,^^9^ Surgical treatment may also be indicated for asymptomatic patients with the objective of averting future complications.^4^ All extrahepatic APFs should be treated, because normally spontaneous closure does not occur.

Patients with refractory symptomatic arterioportal fistulae or who exhibit clinical failure should be treated, with endovascular approaches as the first choice even for high-flow APFs or those with splenic vessel involvement.^4^^,^^7^ Blood flow and fistula diameter are very important to choosing a device that will achieve adequate closure of the communication without embolization of non-target vessels.^4^^,^^10^ Small APFs and those with low blood flow can be adequately treated with embolization agents or sclerosants, whereas APFs will require occlusion devices that are oversized to avoid migration.^4^

Arterial embolization is very effective for treatment of APFs with a single communication, and steel coils, ethanol, embolizing polymer particles, isobutyl cyanoacrylate glue, detachable balloons, occluding devices (vascular plugs), and angioplasty balloons are all used.^4^^,^^7^^,^^11^^-^13 Steel Gianturco type coils are more recommended than embolizing particles because of the risk of unintentional embolization of the terminal splenic circulation.^14^

In cases in which embolization is unsuccessful, surgical treatment with ligature of the hepatic artery or ligature of the fistula are the treatment options.^15^^,^^16^ Splanchnic-portal fistulae can be embolized safely because the spleen has collateral blood supply via the left gastroepiploic artery and the short gastric arteries.^4^ Some groups also recommend complete excision of fistulae as a method of preventing recurrence originating from collateral vessels left untreated when simple ligature of the artery of performed.^17^ The conventional surgical procedure with direct ligature of the fistula and preservation of the arteries involved should be considered the best option for some extrahepatic APFs, for example: APFs involving the superior mesenteric artery, in which embolization or ligature is not recommended, due to the risk of ischemia of the terminal organ, with progression to intestinal necrosis; and APFs that require ligature or embolization of the hepatic artery in patients with limited hepatic functional reserve, with a possibility of liver failure.^1^^,^^18^ In these cases, surgical treatment with direct ligature at the site of fistulization and preservation of the trunk arterial circulation is the better option, but the procedure involves a high level of technical difficulty, primarily because of the increased collateral circulation and the caliber of the veins connected to the fistulae, which can increase the risk of difficult-to-control intraoperative hemorrhages.^18^^,^^19^

However, endovascular embolization of APFs can also involve complications, such as migration of coils, infection, infarction of organs, pancreatitis, and vascular injuries.^19^ The scientific literature on the subject is scant, consisting of retrospective case series or reports of isolated cases, which do not provide high level evidence on the subject. Nevertheless, the results described present functional solutions for selected cases, with low morbidity and mortality.^1^^,^^4^^,^^7^^,^^10^^,^^20^

It can be concluded that for this case of renal-splanchnic fistula with high output and outflow via large caliber veins causing severe PH, endovascular deployment of an oversized vascular occlusion plug proved to be an effective treatment, resulting in significant clinical improvement of the patient.
