# Supplementary material for: Endovascular treatment of portal hypertension and recurrent digestive hemorrhage secondary to arterioportal fistula syndrome: late complication of penetrating abdominal trauma
Source: J Vasc Bras. 2020 Apr 3;19:e20190136. doi: 10.1590/1677-5449.190136 (PMC8202170; doi:10.1590/1677-5449.190136)
Supplement: Translation

# Translation: Tratamento endovascular da hipertensão portal e da hemorragia digestiva recorrente e secundária à síndrome da fístula arterioportal: complicação tardia de trauma abdominal penetrante

**Authors:** Matheus Bertanha, Regina Moura, Rodrigo Gibin Jaldin, Marcone Lima Sobreira, Arthur Curtarelli, Felipe Damacena Rosa, Marcelo Sembenelli, Winston Bonetti Yoshida

**Affiliations:** 1 Universidade Estadual Paulista “Júlio de Mesquita Filho” – UNESP, Cirurgia e Ortopedia, Botucatu, SP, Brasil.; 2 Universidade Positivo, Faculdade de Medicina, Curitiba, PR, Brasil.

**Keywords:** fístula vascular, fístula arteriovenosa, hipertensão portal, ferimentos penetrantes, procedimentos endovasculares

## Abstract

A síndrome da fístula artério-portal (FAP) é uma rara e reversível causa de hipertensão portal pré-sinusoidal, ocasionada pela comunicação de uma artéria visceral com o sistema venoso portal. A maioria dos pacientes é assintomática, mas quando desenvolvem sintomas, estes são mais relacionados com sangramento gastrointestinal, ascite, insuficiência cardíaca congestiva e diarreia. Este desafio terapêutico apresenta um caso de FAP decorrente de ferimento antigo por arma branca e subsequente evolução clínica desfavorável, com grave desnutrição e frequentes hemorragias digestivas. O caso foi solucionado através de oclusão da FAP por meio de tratamento endovascular.

## INTRODUÇÃO

A síndrome da fístula artério-portal (FAP) é uma afecção incomum, frequentemente associada a traumas abdominais, e ocasionada pela comunicação anormal de uma artéria visceral com o sistema venoso portal, resultando em hipertensão portal (HP) pré-sinusoidal^1^. A artéria hepática (65%) e a artéria esplênica (11%), são as artérias mais comumente envolvidas^2^^-^4.

Segundo Guzman et al.^1^, as FAPs podem ser classificadas em três tipos, conforme a etiologia, a anatomia e a topografia:

Tipo 1: constituídas por pequenas fístulas intra-hepáticas periféricas com consequências fisiológicas mínimas, geralmente assintomáticas, e evoluem com oclusão espontânea em 1 mês. Ocorrem frequentemente após biópsias hepáticas percutâneas. Essas fístulas podem ser acompanhadas clinicamente com ultrassom dúplex (USD) por 1 mês, sendo que, se não ocluírem nesse tempo e tornarem-se sintomáticas, devem ser embolizadas;
Tipo 2: são fístulas maiores e mais centrais, intra-hepáticas ou extra-hepáticas. Frequentemente ocorrem após trauma abdominal penetrante ou por erosão de um aneurisma da artéria esplênica e fistulização com o sistema portal. Podem levar a HP, hipertensão hepatoportal e fibrose hepática. Essas fístulas devem ser tratadas com embolização ou por cirurgia convencional (em casos de tratamento endovascular malsucedido ou quando as técnicas endovasculares forem inviáveis), de forma a prevenir as complicações irreversíveis da HP;
Tipo 3: são fístulas congênitas, raras, geralmente intra-hepáticas e difusas, e causam HP grave na infância. Recomenda-se encaminhamento para um centro pediátrico especializado, sendo possíveis as seguintes alternativas terapêuticas: ligadura da artéria hepática, embolização endovascular, hepatectomia ou transplante hepático, a depender do grau de alteração fisiológica.

A sintomatologia das FAPs é variada e depende de vários fatores: diâmetro, topografia e, consequentemente, fluxo sanguíneo da fístula^2^^,^^3^. A maioria dos pacientes com FAP é assintomática, mas quando sintomáticos os pacientes apresentam sangramento gastrointestinal alto (33%), ascite (26%), insuficiência cardíaca congestiva (4,5%) e diarreia (4,5%)^2^. O diagnóstico das FAPs podem ser um desafio, e a arteriografia é o exame de eleição para confirmação^1^. O USD pode ser utilizado como exame de triagem e é um bom exame de seguimento^1^^,^^5^. A angiotomografia computadorizada (angio-TC) pode demonstrar o enchimento precoce de veias na fase arterial do exame e realçar a fase arterial hepática^4^^,^^6^. A angiorressonância magnética pode ser utilizada nos casos em que a arteriografia é contraindicada^4^. A terapia ideal para a FAP permanece controversa. O tratamento específico vai depender do tamanho, da localização e do número de FAPs, além das condições clínicas do paciente e da classificação da doença^7^.

## PARTE I: SITUAÇÃO CLÍNICA

Paciente masculino, 40 anos, foi admitido via pronto-socorro pela equipe de gastroenterologia clínica de nossa instituição em decorrência de hemorragia digestiva alta (HDA) recorrente e vômitos com moderada quantidade de sangue. Apresentava na história clinica episódios frequentes de melena e anorexia; além disso, relatava emagrecimento progressivo. Encontrava-se em mal estado geral, descorado, taquicárdico e normotenso. Negou etilismo, hepatite (cuja ausência foi mais tarde comprovada por sorologias negativas) e outras comorbidades hepatoesplênicas. Ao exame físico, apresentava-se emagrecido, com abdome normal e hepatoesplenomegalia à palpação. Tinha antecedente de trauma abdominal por arma branca em flanco esquerdo há 20 anos. Naquela época, foi submetido a laparotomia exploratória, que resultou somente em nefrectomia esquerda de emergência. Entretanto, evoluiu com HP, tendo sido submetido posteriormente a ligadura endoscópica de varizes esofágicas, para controle de HDA. Há aproximadamente 1 ano, foi submetido em outro serviço a anastomose portossistêmica intra-hepática transjugular (*transjugular intrahepatic portasystemic shunt*, TIPS), porém sem melhora do quadro.

Na entrada em nosso serviço, a investigação complementar com USD mostrou cirrose discreta, pouca quantidade de líiquido livre intracavitário, e aumento do diâmetro da veia porta com fluxo esplênico aumentado, sugestivo de FAP (provável comunicação da artéria renal esquerda residual com a veia esplênica). Esse resultado foi comprovado por angio-TC (Figura 1).

**Figura 1:** Angiotomografia pré-operatória, tempo venoso. (A) renderização volumétrica posteroanterior, demonstrando a artéria renal (seta) e a perfusão precoce das veias esplênica (asterisco), mesentérica superior e porta; (B) mesma imagem com inclinação oblíqua anterior esquerda.

A aortografia diagnóstica comprovou a FAP entre a artéria renal esquerda e a veia esplênica, com alto fluxo sanguíneo, e grande calibre da veia esplênica (Figura 2). Dessa forma, caracterizou-se uma FAP tipo 2 tardia e secundária ao trauma abdominal. A endoscopia digestiva alta identificou varizes de esôfago e fundo gástrico com sinais de sangramento recente, descartando-se úlcera péptica ativa.

**Figura 2:** Aortografia pré-operatória demonstrando o enchimento precoce da veia esplênica (seta).

Frente a esse caso, foram levantadas as seguintes opções de tratamento: tratamento clínico conservador; tratamento cirúrgico convencional com ligadura da artéria renal e esplenectomia; tratamento endovascular com embolização da artéria renal com molas destacáveis; tratamento endovascular com embolização com implante de *plug* vascular destacável.

## PARTE II: O QUE FOI FEITO

O paciente foi submetido ao tratamento endovascular, em que se optou por realizar a oclusão da fístula com implante de sistema de *plug* vascular Cera™ medindo 14 mm × 10 mm (Lifetech Scientific) no coto da artéria renal esquerda. A Figura 3 apresenta maiores detalhes do tratamento, destacando-se a seletivação da artéria renal e a colocação de um introdutor longo (Destination® 8F 65 cm Terumo Medical) (Figura 3A), o posicionamento do sistema de *plug* vascular (Figura 33C), sua liberação após a confirmação do posicionamento e a imagem dos stents do TIPS quase sem contrastação (Figura 3D).

**Figura 3:** Imagens fluoroscópicas do intraoperatório. (A) arteriografia seletiva da artéria renal esquerda (seta) e da fístula com a veia esplênica; (B) posicionamento do *plug* vascular na artéria renal proximal (seta); (C) arteriografia seletiva da artéria renal com o *plug* vascular para checar o posicionamento (seta); (D) aortografia pós-operatória imediata demonstrando o resultado do tratamento, sendo possível visualizar a marcação do *plug* implantado em artéria renal (seta), molas de destaque livre em artéria esplênica (asterisco) e os stents prévios implantados como anastomose portossistêmica intra-hepática transjugular (*transjugular intrahepatic portasystemic shunt*, TIPS) (cabeça de seta).

Como o paciente estava na vigência de hemorragia digestiva por gastropatia hipertensiva, encontrava-se com dificuldade de estabilização dos níveis hematimétricos mesmo com múltiplas transfusões e apresentava plaquetopenia por hiperesplenismo, optou-se por realizar, adicionalmente ao procedimento principal, a embolização da artéria esplênica com duas molas de liberação livre medindo 14 cm × 10 mm Nester® (Cook Medical®), para garantir a redução do débito da veia esplênica, dada a baixa taxa de complicações relacionadas a esse procedimento (Figura 3D). Ao final do procedimento, uma angiografia de controle mostrou lentidão do enchimento do hilo esplênico e oclusão total da artéria renal esquerda, com diminuição significativa do escoamento de contraste para o sistema porta (Figura 3D). O paciente seguiu internado por mais 5 dias para correção da anemia e da desnutrição. Teve melhora significativa do quadro clínico, ganho ponderal, volta às atividades de convívio social e ao trabalho, e está em acompanhamento com USD (6 meses), sem novos episódios de HDA (Figura 4).

**Figura 4:** Ultrassom dúplex de seguimento pós-operatório (6 meses). (A) diâmetro da veia esplênica de 1,47 cm; (B) diâmetro da veia porta de 1,77 cm × 1,80 cm; (C) fluxo venoso monofásico e não fistuloso em veia esplênica; (D) fluxo venoso monofásico em veia porta; (E) fluxo arterial normal em artéria esplênica; (F) fluxo arterial normal em artéria hepática.

## DISCUSSÃO

A minoria dos pacientes portadores de FAP apresenta sintomas e estes, quando ocorrem, são secundários às alterações hemodinâmicas e anatômicas decorrentes do alto fluxo vascular e do aumento da pressão portal^1^. Por outro lado, a ocorrência de HP sem evidência de cirrose pode sugerir FAPs^1^. O diagnóstico pode ser realizado por USD e posteriormente confirmado por angiotomografia ou angiografia seletiva, sendo este o estudo padrão para confirmação diagnóstica e planejamento terapêutico^8^.

A grande maioria dos FAPs não requer intervenção de emergência; há tempo para analisar a fístula e estimar suas consequências fisiológicas; fístulas menores podem sofrer trombose espontânea e, por esse motivo, pequenas fístulas periféricas assintomáticas (classificadas como tipo 1) podem ser acompanhadas com USDs seriados^9^. O tratamento cirúrgico deverá ser indicado se houver evidência de HP, se a fístula for grande, ou se não desaparecer durante um período de tempo não muito longo^4^^,^^9^. O tratamento cirúrgico dos pacientes assintomáticos também pode ser indicado com o objetivo de evitar complicações futuras^4^. Todas as FAPs extra-hepáticas devem ser tratadas, porque normalmente não ocorre fechamento espontâneo.

Pacientes portadores de fístula arterioportal sintomáticos refratários ou em falência clinica devem ser submetidos a tratamento, sendo a modalidade endovascular a primeira escolha mesmo em FAPs de alto fluxo ou que envolvam vasos esplênicos^4^^,^^7^. O fluxo sanguíneo e o diâmetro da fístula têm grande importância na escolha do dispositivo para que se alcance o adequado fechamento da comunicação e não ocorra embolização de vasos não alvo^4^^,^^10^. FAPs pequenas e de baixo fluxo sanguíneo podem ser adequadamente tratadas com agentes embolizantes ou esclerosantes, enquanto as FAPs maiores precisam de dispositivos de oclusão com sobredimensionamento para que não ocorram migrações^4^.

A embolização arterial é muito eficaz no tratamento de FAPs com comunicação única, sendo comumente utilizados molas de aço, álcool etílico, partículas de polímeros embolizantes, cola de isobutil cianocrilato, balões destacáveis, dispositivos oclusores (*plugs* vasculares) e tamponamento com balão de angioplastia^4^^,^^7^^,^^11^^-^13. Molas do aço tipo Gianturco são mais recomendadas que o uso de partículas embolizantes, devido ao risco de embolização não intencional para a circulação esplâncnica terminal^14^.

Em casos onde a embolização não for bem-sucedida, o tratamento cirúrgico com a ligadura da artéria hepática ou a ligadura da fístula são as opções terapêuticas^15^^,^^16^. Fístulas esplenoportais podem ser embolizadas com segurança, porque o baço possui suprimento sanguíneo colateral a partir da artéria gastroepiplóica esquerda e das artérias gástricas curtas^4^. Alguns grupos ainda recomendam a excisão completa das fístulas como forma de prevenir a recorrência proveniente de vasos colaterais não tratados quando se faz a ligadura simples da artéria^17^. O procedimento cirúrgico convencional com ligadura direta da fístula e preservação das artérias envolvidas deve ser considerado a melhor opção para algumas FAPs extra-hepáticas, por exemplo: FAPs que envolvem a artéria mesentérica superior, nas quais a embolização ou a ligadura não é recomendada, devido ao risco de isquemia de órgão terminal com evolução para necrose intestinal; e FAPs que precisariam da ligadura ou embolização da artéria hepática em pacientes com reserva funcional hepática limitada, que poderiam levar a falência hepática^1^^,^^18^. Nesses casos, o tratamento cirúrgico com a ligadura direta do ponto de fistulização e a preservação da circulação arterial troncular é a melhor opção, mas esse procedimento implica em um elevado grau de dificuldade técnica, principalmente pelo aumento da circulação colateral e do calibre das veias conectadas às fístulas, o que pode aumentar o risco de hemorragias de difícil controle no intraoperatório^18^^,^^19^.

Entretanto, a embolização das FAPs via endovascular pode apresentar complicações, como migração de molas, infecção, infarto de órgãos, pancreatite e lesões vasculares^19^. A literatura científica sobre o assunto é escassa, constituída de séries de casos retrospectivas ou relatos de casos isolados, o que não permite maior evidência sobre o assunto. Todavia, os resultados encontrados apresentam soluções funcionais para casos selecionados, com baixa morbimortalidade^1^^,^^4^^,^^7^^,^^10^^,^^20^.

Conclui-se que, para este caso de fístula renoesplênica com alto débito e escape por veias de grande calibre ocasionando grave HP, o implante endovascular de *plug* vascular oclusor sobredimensionado promoveu um tratamento efetivo, com significativa melhora clínica do paciente.
